# Observation of a Long Primo Vessel in a Lymph Vessel from the Inguinal Node of a Rabbit

**DOI:** 10.1155/2013/429106

**Published:** 2013-06-18

**Authors:** Young-Il Noh, Yeong-Min Yoo, Ran-Hyang Kim, Ye-Ji Hong, Hye-Rie Lee, Min-Suk Rho, Sang-Suk Lee

**Affiliations:** ^1^Department of Oriental Biomedical Engineering, College of Health Science, Sangji University, Woosan-dong, Wonju, Gangwon-do 220-702, Republic of Korea; ^2^Department of Biomedical Engineering, College of Health and Science, Yonsei University, Wonju, Gangwon-do 220-710, Republic of Korea; ^3^Department of Oriental Medicine, College of Oriental Medical Science, Sangji University, Woosan-dong, Wonju, Gangwon-do 220-701, Republic of Korea

## Abstract

Though primo vessels are frequently found in the lymph near the abdominal aorta of rabbit by Alcian blue dye, the reproductions are still difficult to require considerable skills and technical know-how at dissected tissue of animal species. However, in the inguinal lymph node of a rabbit we found a long-type primo vascular system (LTP) dyed with Alcian blue, from an abdominal lymph vessel to an inguinal lymph node. The length of LTP was over an average length of 9.1 cm. The average diameters of the primo and the lymph vessels were about 23.9 **μ**m and 242 **μ**m, respectively. The primo vessels were not floating but adhered to lymph vessels with fascial connective tissue. These primo vessels might be a functional integration in the lymph system.

## 1. Introduction

In the history of medical developments, discovery of the new circulating meridian system as blood vessel system and lymphatic system changed the basic paradigm of medicine. Investigation of controller for human body as an autonomic nervous system and a hormone system may be possible to develop the new approach in occurrence and treatment of diseases [1, 2]. In the perspective that the substance of meridian pipe which is path of spirit existed, the two most important things are widely known as spirit and blood in human body to be alive. If an undisclosed structures and functions of the circulating meridian system till now are revealed, it can be an intensely important research that can lead to a revolution in medicine having a bigger destructive power than any medical discovery [3, 4]. 

Recently, the PVS has been found as acupunctural points in mammalian internal organs, such as blood vessels, lymph vessels, spinal cords, brain ventricles, fascia, and skin, by several research groups [5, 6]. Primo vessels have been easily found, by staining with Alcian blue dye to be spread on the surfaces of all the lymphatic vessels [7]. These primo vessels are floating in lymph fluid, and the sanals that exit inside the primo vessels might have a motion property having the cell regeneration [8]. 

Until now, even though intensive research has been dedicated to the PVS during these years, the statistical data on primo vessels in lymph vessel have been available reported. Our group reported already the general morphological features of primo vessels in lymph vessels around the abdominal aorta [9]. The primo vessels in lymphatic vessels were identified from New Zealand white rabbits by micro dissections. 

In this study, we selected lymph vessels around the caudal vena cava connected to lymphatic node in the region neighboring rabbit's inguinal region. This region included many PVS vessels, and those vessels were shown through the reproducible method from among several lymphatic systems. We were able by using the Sangji surgical protocol used on the lymphatic vessels around inguinal region of rabbits during Alcian blue staining to find enough evidence to identify easily the primo vessels under a digital stereo zoom microscope [10]. 

## 2. Materials and Methods

For the laboratory animals, five New Zealand white female rabbits (approximately 1.8 kg) were purchased from Nara-Biotech Animal Company (Seoul, Republic of Korea). All procedures conformed to the ethical regulations for animal experiments constituted by the institutional regulation board of Sangji University (approval number 2012-1). One rabbit was sacrificed for anatomy experiments in the first week, and the other 5 rabbits were sacrificed in one month. Each rabbit was kept in constant temperature and humidity conditions (23°C, relative humidity 60%), with a 12-hour light-dark cycle. All rabbits were deprived of food and water for 1 day before anatomy. 

The rabbits used in the anatomical experiment were sacrificed by injecting 1.5 g/kg of urethane or zolitel intraperitoneally into the peritoneum. The adipose tissues surrounding the inferior vena cava and inguinal region of two legs were then separated and removed. Next, inside the inferior vena cava and inguinal, the PVS, which had been stained blue, was visualized [10]. Images of the PVS under a microscope image analysis system (JSZ-7XT; Samwon, Seoul, Republic of Korea) were captured using a charge coupled device camera (DP70; Olympus, Tokyo, Japan). Other processes of dissection were done with general circumstances of anesthesia [11].

Alcian blue solution was prepared from 0.1 g of Alcian blue (Sigma, St. Louis, MO, USA) in 10 mL of phosphate-buffered saline (PBS, pH 7.4) and was filtered by using a 0.45 *μ*M membrane filter (Merck Millipore, Darmstadt, Germany) with a syringe (BD, Franklin Lakes, NJ, USA). After the sides of rabbit's inguinal region in part of two legs had been incised, Alcian blue solution, preheated to 37°C in a water bath, was injected into inguinal lymph bundles.

## 3. Results and Discussion

**Figure 1:**
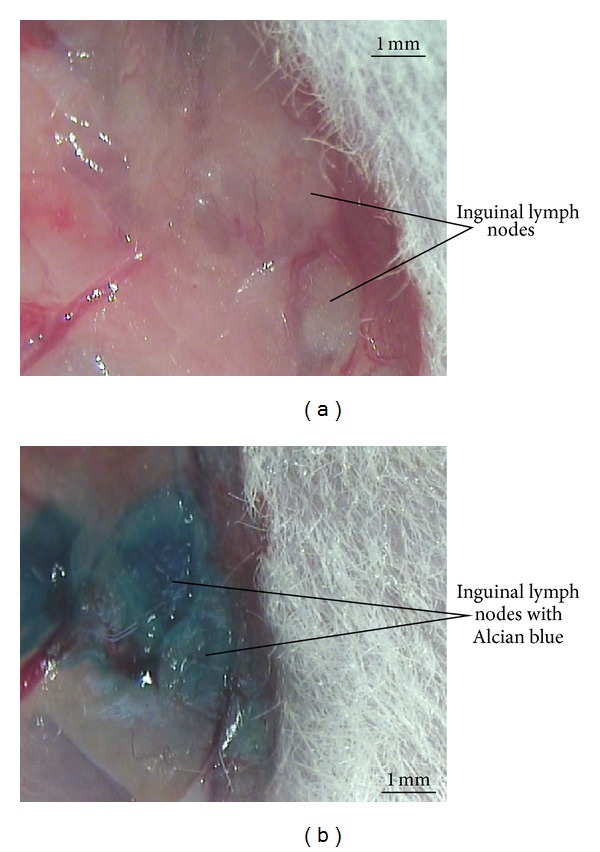
Rabbit's inguinal lymph nodes before (a) and after (b) Alcian blue injection.

For easy reproducible isolation, we chose inguinal lymphatic vessels but did not choose abdominal lymph vessel and dark-red blood vessel, and Alcian blue staining was required for transparent lymphatic vessels. We attempted to identify large inguinal lymph vessels in the region neighboring the caudal vena cava with a digital stereo microscope. Alcian blue solution, 200 *μ*L, was injected into inguinal lymph nodes that included a lymph vessel for *in situ* visualization of PVS's vessels [12]. Figures 1(a) and 1(b) show rabbit's inguinal lymph bundles before and after Alcian blue injection.

**Figure 2:**
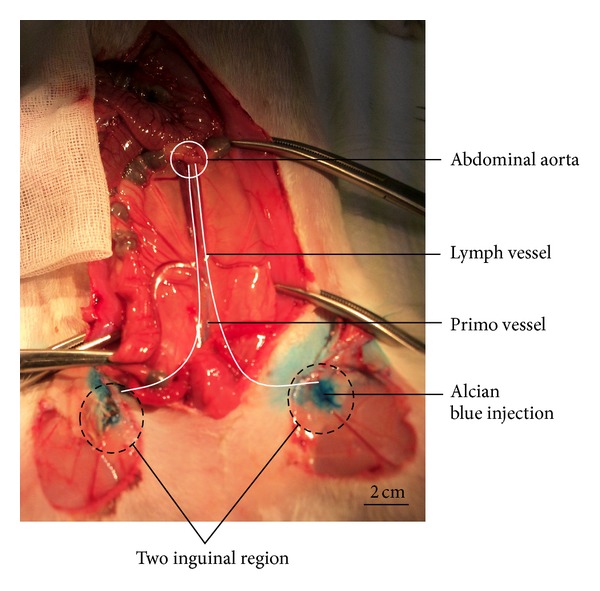
PVS vessel point from lymph nodes of two inguinal regions to lymph vessel of vena cava of a rabbit by injection of Alcian blue.

To investigate primo vessels from an abdominal lymph vessel to an inguinal lymph node, we injected Alcian blue into two inguinal lymph nodes. Immediately Alcian blue solution flowed into inguinal lymph vessel and arrived at an abdominal lymph vessel. This indicates that the primo vessels may be a vascular system connect an abdominal lymph node to an inguinal lymph node, as shown in Figure 2.

**Figure 3:**
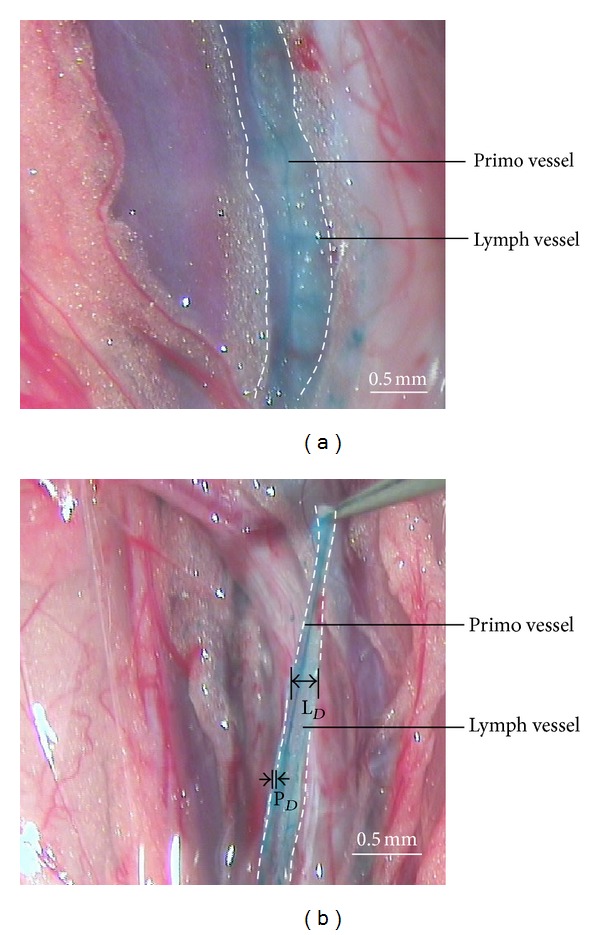
PVS vessels are active on the rabbit's respiration. Lymphatic primo vessels attached organs stained by Alcian blue inside the lymph vessels (a). The isolated primo vessel with lymph vessel from organ (b). White dot lines are lymph vessels.

Figure 3(a) shows lymphatic primo vessels attached organs stained by Alcian blue inside lymph vessels.  Figure 3(b) shows the isolated primo vessel inside lymph vessel. The primo vessel inside the lymph vessel as a strand-like microtubular PVS stained with Alcian blue and is floating inside a lymph vessel. PVS vessels are a living tissue in the rabbit's respiration. We already identified PVS which has rod-shaped nuclei with DAPI in a previous report [9].

Table 1 shows data analysis of the morphological features, including the diameters of the lymph vessels and primo vessels observed from the 5 samples of primo vessels in lymph vessels. The number of primo vessels and the average diameter and length of a primo vessel with standard deviation are also shown in Table 1. The subject numbers are ordered according to the dates on which the experiments were performed. The observation of the diameter of a lymph vessel (*L*_*D*_) and the diameter of a primo vessel (*P*_*D*_) are shown in Figure 3.

Of the 5 lymph vessels observed, all had primo vessels. The average diameter of the lymph vessels inside the caudal vena cava of the 5 rabbits was 242 *μ*m. This result is almost uniform as the size of the lymph vessels. Also, the average diameter of the primo vessels was 23.9 *μ*m. The average diameters of the lymph vessels and the primo vessels agreed with the sizes of the lymph vessels and the primo vessels, respectively, which are similar to previously reported values (20 *μ*m~30 *μ*m) [9, 11, 13, 14]. 

We already reported that the microdissected specimens *in situ* reveal rod-shaped nuclei stained by acridine orange. Also, the blue-stained nuclei having a broken-lined stripe and a tube structure and the distance between the nuclei of two cells on neighboring aligned stripes were measured to be about 20 *μ*m in diameter and about 5 *μ*m~10 *μ*m, respectively [9].

There are some of the remaining debris that still adhered to the primo vessels in the process of this experiment. The average length of the primo vessels from the lymph vessels was 9.1 mm, and this was fairly uniform throughout the samples and longer than what several primo research groups found depending on the lymph vessels and the physiological state of the subject.

**Figure 4:**
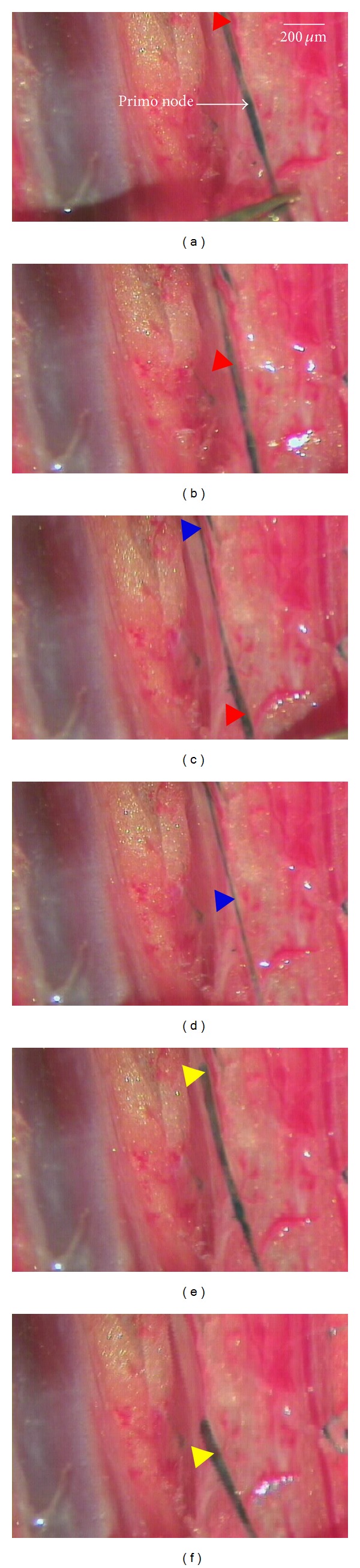
Visualization of the extraction process for a primo vessel inside the lymph vessel attached to node in the inguinal lymph of a rabbit. A thick primo vessel indicated by arrows in status before (a) and after (b) extraction by using micro-pincett. Images of two steps ((c), (d)) during the extraction of a very slender primo vessel inside the lymph vessel. Images of two steps ((e), (f)) during the extraction of a thick primo vessel indicated by arrows in status extraction by using micro-pincett.

Figures 4(a), 4(b), 4(c), 4(d), 4(e), and 4(f) show steps of the extraction process for a primo vessel inside the lymph vessel connected to node in the inguinal lymph of a rabbit. First, Figures 4(a) and 4(b) show images of a thick primo vessel indicated by arrows in status before and after extraction by using tweezers, respectively. Second, Figures 4(c) and 4(d) show images of two steps during the extraction of a very slender primo vessel inside the lymph vessel with pincett. The presence of the Bonghan system is hardly noticeable. Finally, Figures 4(e) and 4(f) show images of two steps during the extraction of a thick primo vessel indicated by arrows in status extraction by using micro-pincett. There is a long branching of the thin and thick primo vessels inside the lymph vessel having an average length above 9.1 cm. It is also significant that the primo vessel exists in the lymph attached to nearby two inguinals. The primo vessels were not floating but adhered to lymph vessels with fascial connective tissue. These attached primo vessels might be a functional integration in the lymph system [12, 15, 16].

## 4. Conclusion

We demonstrate that primo vessels in lymphatic vessels around the abdominal aorta connected to inguinal lymph nodes of rabbits can be simply identified under a digital stereo microscope by using Alcian blue staining. In the inguinal lymph node of a rabbit, we found a long-type primo vascular system. The length of LTP was over an average length of 9.1 cm. The average diameters of the primo and the lymph vessels were about 23.9 *μ*m and 242 *μ*m, respectively. The primo vessels were not floating but adhered to lymph vessels with fascial connective tissue. These adhering primo vessels might be an integrated functional system in lymph. The molecular functions and electrical characteristics of primo vessels in lymph vessels may open a new approach to treating chronic diseases such as cancer, diabetes, and cerebral apoplexy, to wound healing, and to acupuncture meridian medicine in particular. 

## Figures and Tables

**Table 1:** Morphological analysis data of the PVS and the lymph vessels near the abdominal aorta connected to inguinal of five rabbits.

Subject number	Sex	Weight (kg)	Lymph vessel			Primo vessel		
			PV	LV	*L*_*D*_ (*μ*m)	PN	*P*_*D*_ (*μ*m)	*l* (cm)
1	F	1.9 kg	O	O	230	6	23.1	8.0
2	F	1.8 kg	O	O	255	8	22.5	9.5
3	F	1.8 kg	O	O	245	8	23.8	8.4
4	F	1.8 kg	O	O	255	6	24.9	9.5
5	F	1.9 kg	O	O	225	7	25.6	10.1

Ave.					242		23.9	9.1
S.D.					12.4		1.1	0.7

PV: primo vessel; LV: lymph vessel; *L*_*D*_: diameter of lymph vessel; PN: number of primo nodes; *P*_*D*_: diameter of primo vessel; *l*: length of primo vessel; Ave.: average value; S.D.: standard deviation.

## References

[1] Jeltsch M, Kaipainen A, Joukov V (1997). Hyperplasia of lymphatic vessels in VEGF-C transgenic mice. *Science*.

[2] Lee B-C, Yoo JS, Baik KY, Kim KW, Soh K-S (2005). Novel threadlike structures (Bonghan ducts) inside lymphatic vessels of rabbits visualized with a Janus Green B staining method. *Anatomical Record B*.

[3] Kwon J, Baik KY, Lee B-C, Soh K-S, Lee NJ, Kang CJ (2007). Scanning probe microscopy study of microcells from the organ surface Bonghan corpuscle. *Applied Physics Letters*.

[4] Lee B-C, Soh K-S (2008). Contrast-enhancing optical method to observe a Bonghan duct floating inside a lymph vessel of a rabbit. *Lymphology*.

[5] Kim BH (1962). Study on the reality of acupuncture meridians. *Journal of Jo Sun Medicine*.

[6] Kim BH (1965). Sanal theory. *Journal of Jo Sun Medicine*.

[7] Kim BH (1963). On the Kyungrak system. *Journal of the Academy of Medical Sciences of the Democratic People's Republic of Korea*.

[8] Soh K-S (2004). Bonghan duct and acupuncture meridian as optical channel of biophoton. *Journal of the Korean Physical Society*.

[9] Noh YI, Rho M, Yoo YM, Jung SJ, Lee SS (2012). Isolation and morphological features of primo vessels in rabbit lymph vessels. *Journal of Acupuncture and Meridian Studies*.

[10] Lee B-C, Kim KW, Soh K-S (2009). Visualizing the network of bonghan ducts in the omentum and peritoneum by using trypan blue. *Journal of Acupuncture and Meridian Studies*.

[11] Lee B-C, Yoo JS, Ogay V (2007). Electron microscopic study of novel threadlike structures on the surfaces of mammalian organs. *Microscopy Research and Technique*.

[12] Yoo JS, Kim HB, Won N (2011). Evidence for an additional metastatic route: in vivo imaging of cancer cells in the primo-vascular system around tumors and organs. *Molecular Imaging and Biology*.

[13] Sung B, Kim MS, Lee B-C (2008). Measurement of flow speed in the channels of novel threadlike structures on the surfaces of mammalian organs. *Naturwissenschaften*.

[14] Ogay V, Bae KH, Kim KW, Soh K-S (2009). Comparison of the characteristic features of Bonghan ducts, blood and lymphatic capillaries. *Journal of Acupuncture and Meridian Studies*.

[15] Yoo JS, Ayati MH, Kim HB, Zhang W-B, Soh K-S (2010). Characterization of the primo-vascular system in the abdominal cavity of lung cancer mouse model and its differences from the lymphatic system. *PLoS One*.

[16] Kato S, Shimoda H, Ji R-C, Miura M (2006). Lymphangiogenesis and expression of specific molecules as lymphatic endothelial cell markers. *Anatomical Science International*.

